# A clinical study of ultrasound-guided acupotomy combined with mindfulness meditation to improve lumbar myofascial pain syndrome

**DOI:** 10.3389/fneur.2025.1627376

**Published:** 2025-10-02

**Authors:** Yujia Liu, Jiayi Shang, Yuxuan Zhang, Li Liu, Wenbing Zhang

**Affiliations:** ^1^Department of Rehabilitation, Pingtan Comprehensive Experimental Area Hospital, Fuzhou, China; ^2^Fujian University of Traditional Chinese Medicine, Fuzhou, China; ^3^Department of Painology, Fujian University of Traditional Chinese Medicine Affiliated People’s Hospital, Fuzhou, China

**Keywords:** lumbar myofascial pain syndrome, ultrasound-guided, acupotomy, mindfulness meditation, MPS

## Abstract

**Objective:**

To evaluate the efficacy and safety of ultrasound-guided acupotomy combined with mindfulness meditation for the treatment of lumbar myofascial pain syndrome (MPS).

**Methods:**

This blinded randomized controlled trial lasted for 3 weeks and included a 90-day follow-up. The participants were 120 patients with lumbar MPS. These patients were randomized into three groups: Group A (ultrasound-guided acupotomy combined with mindfulness meditation, *n* = 40), Group B (ultrasound-guided acupotomy, *n* = 40), and Group C (celecoxib, *n* = 40). Data were collected at baseline, week 1, week 2, week 3 (posttreatment) and day 90 (follow-up).

**Results:**

Group A was superior to Group B and significantly superior to Group C in terms of pain level (measured via the McGill Pain Questionnaire), lumbar spine mobility and mood disorders (measured via the Hospital Anxiety and Depression Scale), and quality of sleep (measured via the Pittsburgh Sleep Quality Index) (*p* < 0.05). Furthermore, the treatment efficacy was more durable in Group A (there was no significant rebound at 90 days of follow-up). The TNF-α and IL-1β serum levels were reduced in all three groups at week 3 but were more pronounced in the celecoxib group. Compared with ultrasound-guided acupotomy and celecoxib, ultrasound-guided acupotomy combined with mindfulness meditation can rapidly relieve pain, improve lumbar spine function, and permanently improve patients’ psychological state and sleep quality through the dual mechanism of “peripheral relaxation-central regulation.” This approach can treat the lumbar MPS from the root and provides new ideas for the clinical diagnosis and treatment of chronic pain.

## 1 Introduction

Lumbar myofascial pain syndrome (MPS) is a common chronic musculoskeletal pain disorder that is characterized by localized muscle pressure points and tenderness (1). Patients with low back pain have palpable painful striated nodules called “myofascial trigger points (MTrPs)” on skeletal muscles, which trigger pain and localized twitching responses to pressure (2). Furthermore, these patients may also cause distal referred pain. Previous studies revealed that approximately 44% of adults report chronic musculoskeletal pain, of which lumbar MPS is one of the main causes (3–5). This disease not only causes physical pain in patients but is also often accompanied by autonomic abnormalities, as well as psychological problems such as sleep disorders, anxiety and depression, which seriously affect patients’ social functioning and work efficiency (6, 7). Therefore, it is necessary to develop methods for alleviating psychological problems, reducing pain and improving quality of life among lumbar MPS patients.

Traditional treatments for lumbar MPS mainly include medication, physical therapy and exercise therapy (8). Pharmacological treatments commonly use nonsteroidal anti-inflammatory and analgesic drugs, antidepressants, sodium/calcium ion pathway regulators, muscle relaxants, and various types of opioids, but long-term use of these drugs may lead to gastrointestinal reactions and injuries, liver and renal impairment and other side effects (9). Furthermore, lumbar MPS is prone to recurrence after drug cessation. Physical therapies, such as heat therapy, shock wave therapy, and phototherapy, require long-term adherence and are characterized by poor patient compliance and certain limitations in therapeutic effects. Exercise therapy is widely recommended, but it imposes certain requirements on the patient’s basic health condition and may be difficult to implement in patients with severe pain (1).

As a product of the combination of traditional Chinese medicine and modern medicine, acupotomy therapy has received an increasing amount of attention in the field of chronic pain management in recent years. Acupotomy exerts analgesic effects through mechanisms such as loosening local muscle adhesions, relieving muscle spasms and regulating neurotransmitters (10, 11). The introduction of ultrasound-guided technology provides precise imaging support for acupotomy treatment, which can reveal the anatomical structure of muscles, fascia and MTrPs in real time, making up for the inaccuracy of localization and depth of needling that may exist in traditional acupotomy treatment, which relies on the experience and palpation of the doctor (12, 13). Therefore, ultrasound-guided acupotomy leads to improvements in accuracy and safety. However, the role of a single ultrasound-guided acupotomy in improving patients’ psychological status and quality of life remains unclear.

Meditation, which is a method of mind–body regulation that began more than 3,000 years ago, has gained an increasing amount of attention worldwide. Furthermore, the effects of meditation have been corroborated by objective data such as *in vivo* encephalography, physiological scales, and laboratory reports (14–16). Mediation has also attracted an increasing amount of attention in the field of chronic pain management in recent years. Studies have shown that meditation can regulate the function of the autonomic nervous system, reduce the stress response, improve pain cognition, and thus alleviate chronic pain (14, 17, 18). In particular, mindfulness meditation has been shown to significantly improve the psychological state and quality of life of chronic pain patients (19, 20). However, further research is necessary to elucidate the mechanism of action underlying mindfulness meditation as a psychological intervention in relieving somatic symptoms.

On the basis of the above background, the aims of this study were to investigate the clinical efficacy of ultrasound-guided acupotomy combined with mindfulness meditation for the treatment of lumbar MPS; to determine whether the combination of these two methods can produce synergistic effects; and to provide comprehensive interventions for lumbar MPS at both the physiological and psychological levels to alleviate pain, improve lumbar mobility, enhance quality of life, and promote psychological health. This study can help improve the therapeutic means of treating lumbar MPS and provide new ideas for comprehensive clinical diagnosis and treatment.

## 2 Materials and methods

### 2.1 Study design

This study was a randomized controlled trial (RCT) with a single-blind design. The clinical trial lasted for 3 weeks and included a 90-day follow-up assessment. The participants were 120 patients with lumbar myofascial pain syndrome in the Department of Painology, Fujian University of Traditional Chinese Medicine Affiliated People’s Hospital. The aim of this RCT was to evaluate the effectiveness and safety of ultrasound-guided acupotomy combined with mindfulness meditation in terms of improving pain, function, mood disorders, and sleep quality and inflammatory factors in patients with lumbar MPS. All participants provided oral and written informed consent prior to study enrolment. This study was conducted according to the CONSORT statement. All the subjects signed a written informed consent form prior to study enrolment. The study was approved by the Ethical Review Committee of Fujian University of Traditional Chinese Medicine Affiliated People’s Hospital.

### 2.2 Participants

The inclusion criteria were as follows: (1) met the diagnostic criteria for lumbar MPS; (2) had a visual analogue scale (VAS) score >3; (3) complained of low back pain for more than 3 months; (4) aged between 18 and 60 years; (5) did not receive any other therapeutic methods or oral analgesic, antidepressant or anti-insomnia treatments within 3 months; and (6) provided informed consent.

The exclusion criteria were as follows: (1) contraindications to celecoxib; (2) inability to undergo acupotomy treatment; (3) severe coagulation disorders; (4) ankylosing spondylitis, lumbar spine fracture, dislocation, or herniated intervertebral disks; (5) abnormalities in lumbosacral nerve function; (6) pregnancy or breastfeeding; (7) cognitive dysfunction; (8) localized skin rupture or erythema; (9) rheumatoid disorders; (10) a combination of cardiac, cerebral, hepatic, renal, and other serious organ diseases or mental diseases; (11) receiving mindfulness meditation therapy; or (12) liver, kidney, and other serious organ diseases or mental illness.

### 2.3 Randomization and blinding

A total of 120 participants were randomly assigned in a 1:1:1 ratio to one of three groups: the ultrasound-guided acupotomy combined with mindfulness meditation group (*n* = 40), the ultrasound-guided acupotomy group (*n* = 40), or the oral celecoxib group (*n* = 40). The random allocation sequence was generated by an independent statistician using SPSS 26.0, and sealed in opaque, sequentially numbered envelopes. These envelopes were prepared by a study coordinator who was not involved in participant recruitment, treatment administration and outcome assessment. Upon participant enrollment, the coordinator opened the corresponding envelope and disclosed the group allocation only to the relevant intervention provider. Group allocation was displayed only when patients were enrolled to ensure allocation concealment. The three treatment groups were conducted in different clinics, with intervention providers delivering only their own intervention protocols and not cross-participating in other groups’ interventions. During the study, participants were told not to disclose their treatment to avoid detection bias.

This study adopted a single-blind design in which only the outcome assessors and data managers were blinded to group allocation. The substantial differences in treatment procedures among acupotomy, meditation, and oral celecoxib administration rendered blinding of both participants and practitioners were unfeasible. To minimize potential bias, different personnel were assigned for treatment delivery, outcome assessment, data management, and statistical analysis. Each intervention provider was limited to single modality and was excluded from outcome evaluation.

### 2.4 Interventions

**Figure 1:**
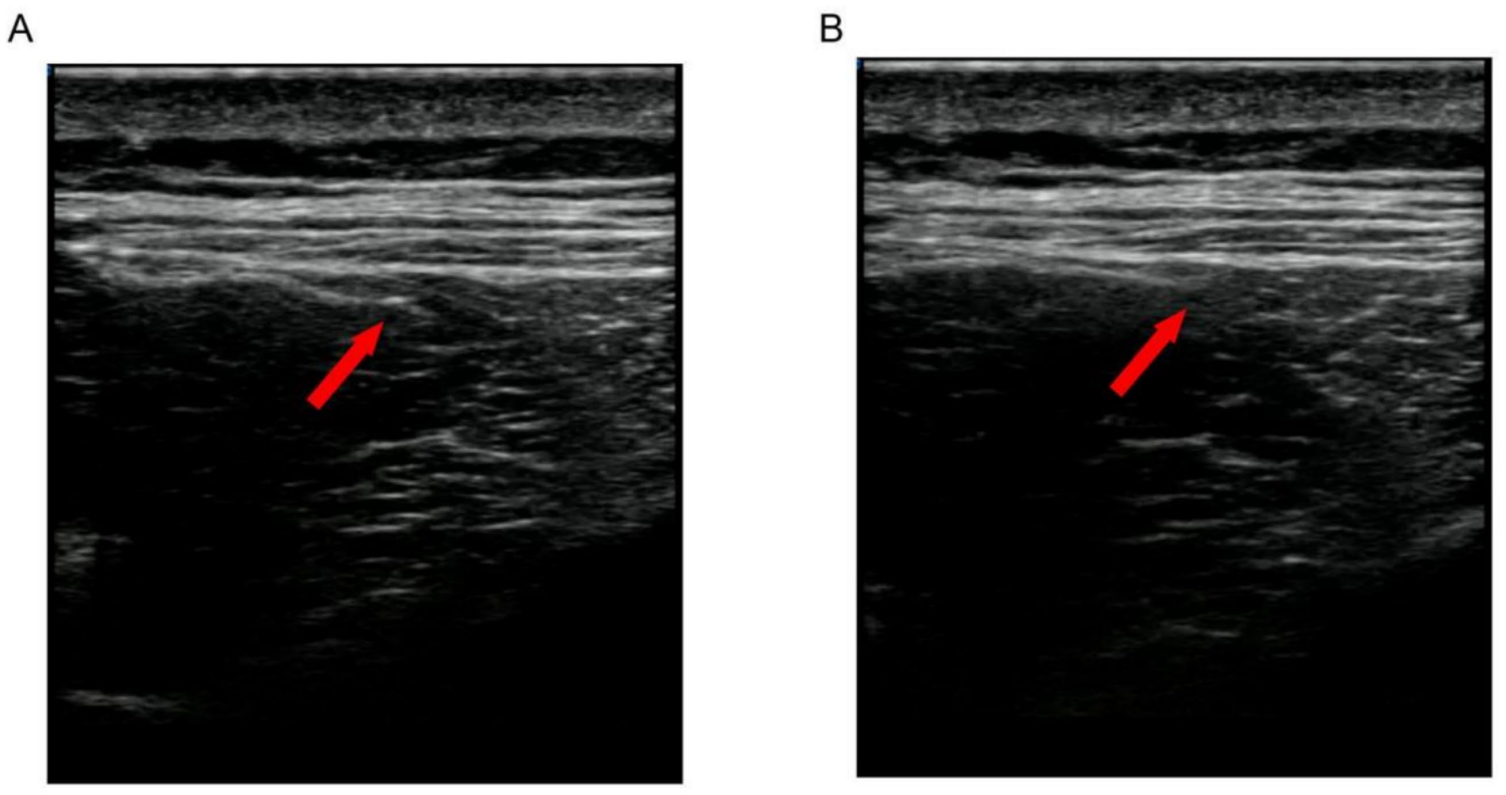
Two-dimensional ultrasonography images from the same patient before and after treatment. The red arrow: myofascial trigger points (MTrPs). **(A)** MTrPs showing oval, locally heterogeneous hyper echoic areas in two-dimensional ultrasound observation. **(B)** After repeated puncture of the MTrPs with a dry needle, the localized echoes diminished. With a dry needle, the localized echoes diminished, and the entangled muscles unraveled and relaxed.

In Group B (the ultrasound-guided acupotomy group), (1) the patient was placed in a prone position, with the treatment area fully exposed; the doctor was located on the patient’s side; and the ultrasound instrument was placed in front of the doctor. (2) Palpation and localization: The lumbar erector spinae and multifidus muscles were palpated and localized by the same experienced clinician, and each subject was palpated bilaterally next to the spinous processes of the lumbar vertebrae L3 and L5. When pressure points and mass-like painful hard nodules or striated muscle fiber spasm bands were palpated, MTrPs were marked with a pen at the site. (3) Disinfection: A 15-cm area centered on the fixation point was disinfected with iodine volts 2 times, after which a sterile cavity towel was spread out. (4) Ultrasound-guided acupotomy manipulation: A SONI MAGE HS1 color ultrasonography diagnostic instrument was used with an ultrasound probe model number of L11-3, and longitudinal cuts and transverse cuts were made along the lumbar erector spinae muscle fibers, and the lumbar spine muscle fibers were cut longitudinally and transversally. Longitudinal and transverse scans were performed along the lumbar erector spinae muscle fibers to confirm the extent of the erector spinae and multifidus muscles as well as the lumbar spinous processes and articular syndromes and to search for MTrPs. Normal muscle fibers were hypoechoic under ultrasound imaging, and MTrPs were oval shaped, locally heterogeneous and hyperechoic areas. Under ultrasound guidance, the acupotomy (0.6 × 50 mm, Beijing Huaxia Acupotomy Medical Instrument Factory) was inserted into the muscle tissue with the knife line parallel to the longitudinal axis of the torso and the body of the acupotomy perpendicular to the ketosis body, and two to three cuts were made into each area until the abnormal echoes in that area were not visible (Figure 1). If there are multiple MTrPs, the above treatment should be repeated. (5) Postoperative treatment: Pressure was applied to stop the bleeding, and a band aid was applied. Treatments were performed once a week for 15 min, three times per course of treatment, for a total of 3 weeks.

In Group A (ultrasound-guided acupotomy + mindfulness meditation group), ultrasound-guided acupotomy was performed using the same method as in Group B. Mindfulness meditation training was led by a professional MBSR instructor. (1) Breathing meditation (basic training): Patients sat in a comfortable position, closed their eyes and focused on the rhythm of natural breathing. When attention was distracted by pain or distractions, the patient was encouraged to gently redirect their attention to breathing. (2) Body scanning (targeted pain management): The patient was guided to start at the feet and gradually scan the entire body (focusing on painful areas). Encourage the patient to observe the sensations (e.g., burning and tightness) in the painful area with an attitude of “curiosity” rather than “resistance.” Imagine “bringing the breath into the painful area” in conjunction with breathing to promote relaxation. (3) Positive walking (dynamic training): The patient walked slowly, focusing on the sensation of the soles of the feet touching the ground and the contraction and relaxation of the muscles. When pain appeared, the patient was guided to pause and observe the change in pain instead of rushing to escape. (4) Emotion regulation (cognitive restructuring): Emotion regulation helps patients recognize pain-induced emotions (e.g., fear, anger) through guided speech. Patients practiced accepting emotions from a “bystander” perspective rather than by confronting them. Meditation effectiveness was scored weekly using the Five-Facet Mindfulness Questionnaire (FFMQ) on the day of ultrasound-guided acupotomy. Treatments were given three times per week for 30 min each and during each course of the treatment consisted of nine treatments for a total of 3 weeks.

Group C underwent oral celecoxib treatment, including oral celecoxib capsules (celecoxib, Jiangsu Daqing Pharmaceutical Factory, National Drug Code: H20193414, 200 mg), 200 mg once daily for 3 weeks.

### 2.5 Outcome

The participants completed questionnaires and inflammation level tests before treatment and on days 7, 14, 21, 90. Observational indicators: ① Pain level: Pain was evaluated by the McGill Pain Questionnaire (SF-MPQ), which consists of three dimensions: the pain rating index (PRI) (0 = none, 1 = mild, 2 = moderate, 3 = severe), the visual analogue scale (VAS) (score of 0–10, in which 0 = none pain, and 10 = worst pain), and the present pain intensity (PPI) (score of 0–5), in which 0 = (0–5, where 0 = no pain, 5 = unbearable pain). Higher scores indicate more severe pain. ② Lumbar spine activity function: The angles of lumbar spine rotation during forwards flexion, backwards extension, left lateral flexion and right lateral flexion were measured by a protractor to assess the patient’s lumbar spine activity function. Larger angles indicated better lumbar spine activity function. ③ Mood disorders and sleep quality were evaluated by the Hospital Anxiety and Depression Scale (HADS), which includes 14 items across two subscales: anxiety (HADS-A) and depression (HADS-D). Each subscale includes seven items. The total score ranges from 0 to 21 points, with higher scores indicating more severe mood disorders. Sleep quality was evaluated using the Pittsburgh Sleep Quality Index (PSQI), which assesses seven aspects of sleep disorders, including time to sleep, hypnotic medication, sleep duration, sleep efficiency, sleep quality, and daytime activities. Each aspect is rated on a scale ranging from 0 to 3 points, and the total score ranges from 0 to 21 points, with higher scores indicating poorer sleep quality. ④ Tumor necrosis factor alpha (TNF-α) and interleukin-1β (IL-1β): Serum levels of TNF-α and IL-1β were measured by enzyme-linked immunosorbent assay (ELISA) with strict reference to the instructions of the kit, and higher serum levels indicated greater expression of inflammatory factors and stronger inflammatory responses.

### 2.6 Follow-up protocol

To maintain a high retention rate of follow-up participants, the following measures were taken: the investigators made weekly contact with participants via phone calls or text message during the study to check on their current condition and inform them of the specific time for the follow-up assessment. A staff member was designated to manage follow-up activities, record participants’ responses, and promptly address any concerns or barriers that could affect continued participation. The research team also collected alternate contact information for each participant to ensure timely communication. Additionally, assessments of pain level, psychological status, and sleep quality could be completed via phone calls or text message. These strategies contributed to achieve a 90 percent retention rate at follow-up.

### 2.7 Statistical analysis

Statistical analysis was performed using SPSS 26.0. The measurement data are expressed as the mean ± standard deviation ( $\bar{x}\;$ ± s). Comparisons between groups were conducted by ANOVA, comparisons at different time points were performed via repeated-measures ANOVA, and comparisons of count data were performed by the *χ*^2^ test. *p* < 0.05 indicated a statistically significant difference.

## 3 Results

### 3.1 Comparison of the general characteristics of the three groups of patients

**Figure 2:**
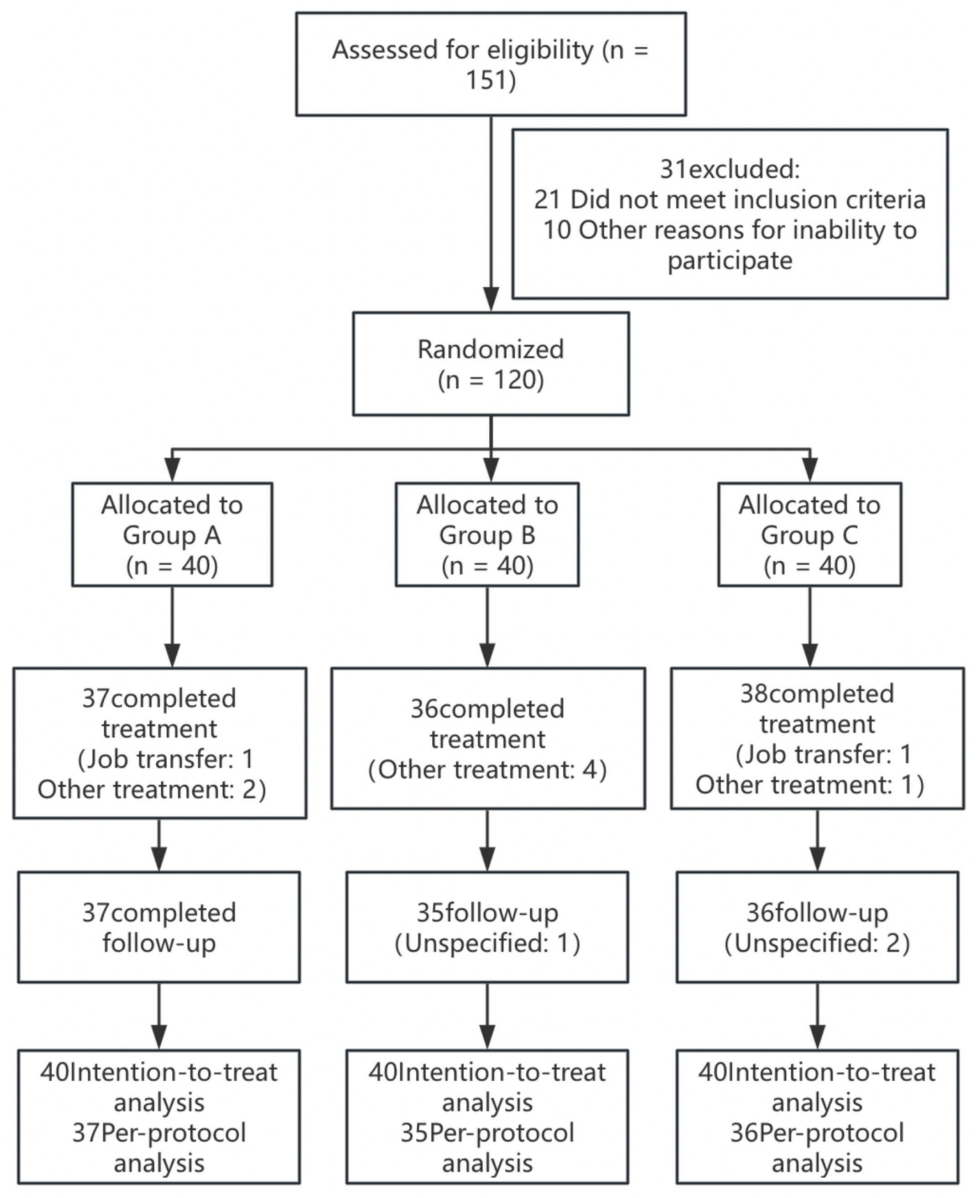
Patient enrollment flowchart.

There was no difference in sex, age, duration of disease, or education among the three groups (*p* > 0.05) (Table 1). Between February 2025 and May 2025, 120 lumbar MPS patients were randomly assigned to Group A (*n* = 40; men = 19, women = 21), Group B (*n* = 40; men = 20, women = 20), or Group C (*n* = 40; men = 18, women = 22), with the last participant completing the follow-up visit in May 2025. A total of 111 patients (93%) completed the treatment (two patients withdrew due to job transfer, and seven patients withdrew due to other treatment interventions), and 108 patients (90%) completed follow-up (Figure 2). Two patients in Group A and 1 patient in Group B experienced localized bleeding at the acupotomy site, which ceased after 5 min of pressure. A total of 108 patients had no other adverse events.

**Table 1:** Comparison of the general characteristics of the three groups of patients.

Group	*N*	Sex		Average age	Average course of disease	Education	
		Male	Female	( $\bar{x}\;$ ± s, years)	( $\bar{x}\;$ ± s, years)	Primary and below	Junior high school and above
A	40	19	21	44.73 ± 6.21	5 ± 1.88	14	26
B	40	20	20	45.66 ± 5.92	5 ± 2.10	14	26
C	40	18	22	46.66 ± 4.92	5 ± 2.33	12	28

### 3.2 Comparison of pain scores (SF-MPQ scores) among the three groups at different time points

In this study, the SF-MPQ scores for each dimension of lumbar MPS were compared across three groups of patients (Table 2). Before treatment (T0), there was no significant difference in the SF-MPQ scores for each dimension among the three groups (*d* < 0.2, *p* > 0.01), indicating that the baseline pain levels were comparable. During the 21-day treatment period, Group A demonstrated a rapid onset of action: the PRI score decreased from 25.3 ± 4.2 to 8.2 ± 2.8 (67.6% decrease), the VAS score from 7.2 ± 1.5 to 3.0 ± 0.9 (58.3% decrease), and the PPI score from 3.8 ± 0.8 to 1.5 ± 1.4 (60% decrease). Group B also showed significant efficacy: the PRI score decreased from 24.8 ± 3.9 to 9.8 ± 3.2 (60.5% decrease), the VAS score decreased from 7.3 ± 1.4 to 3.6 ± 1.0 (50.7% decrease), and the PPI score decreased from 3.7 ± 0.7 to 1.8 ± 0.5 (51.4% decrease). At AT21, the effect sizes (Cohen’s *d*) for comparisons between Group A and Group C were −1.41 for PRI, −1.00 for VAS, and −2.86 for PPI. In comparison, the effect sizes between Group B and Group C were −0.83, −0.38, and −1.71, respectively. These findings indicate that both Group A and Group B achieved significantly greater reductions in pain scores compared to Group C (*p* < 0.05), with Group A demonstrating a more substantial and clinically meaningful improvement.

**Table 2:** Comparison of pain scores (SF-MPQ scores) among the three groups at different time points.

SF-MPQ	Time	Group A (*n* = 40)	Group B (*n* = 40)	Group C (*n* = 40)	Cohen’s *d* A vs. B	Cohen’s *d* A vs. C	Cohen’s *d* B vs. C
PRI	T0	25.3 ± 4.2	24.8 ± 3.9	25.1 ± 4.0	0.12	0.05	−0.08
	AT7	18.5 ± 3.5^bc^	20.2 ± 3.7^c^	21.8 ± 3.4	−0.47	−0.96	−0.45
	AT14	12.5 ± 3.1^bc^	15.3 ± 3.4^c^	18.7 ± 3.2	−0.86	−1.96	−1.03
	AT21	8.2 ± 2.8^bc^	9.8 ± 3.2^c^	12.5 ± 3.3	−0.53	−1.41	−0.83
	AT90	8.8 ± 2.8^bc^	11.0 ± 3.2^c^	20.1 ± 3.1	−0.73	−3.83	−2.89
VAS	T0	7.2 ± 1.5	7.3 ± 1.4	7.1 ± 1.6	−0.07	0.06	0.13
	AT7	5.1 ± 1.2^bc^	5.6 ± 1.3^c^	5.8 ± 1.2	−0.40	−0.58	−0.16
	AT14	3.3 ± 1.0^bc^	4.2 ± 1.1^c^	4.8 ± 1.3	−0.86	−1.29	−0.50
	AT21	3.0 ± 0.9^bc^	3.6 ± 1.0^c^	4.0 ± 1.1	−0.63	−1.00	−0.38
	AT90	3.1 ± 0.9^bc^	3.9 ± 1.0^c^	5.5 ± 1.4	−0.84	−2.05	−1.31
PPI	T0	3.8 ± 0.8	3.7 ± 0.7	3.8 ± 0.9	0.13	0.00	−0.12
	AT7	2.5 ± 0.6^bc^	2.7 ± 0.7^c^	3.3 ± 0.2	−0.31	−1.78	−1.18
	AT14	1.8 ± 0.5^bc^	2.0 ± 0.6^c^	2.8 ± 0.7	−0.36	−1.64	−1.23
	AT21	1.5 ± 0.4^bc^	1.8 ± 0.5^c^	2.5 ± 0.3	−0.67	−2.86	−1.71
	AT90	1.6 ± 0.4^bc^	2.0 ± 0.4^c^	2.9 ± 0.4	−1.00	−3.22	−2.24

vs. Group B, ^b^*p* < 0.05; vs Group C, ^c^*p* < 0.05. All *p*-values for time, between groups, and time × group interactions were <0.05.

The results of the follow-up assessment (AT21–AT90) revealed that the scores of Group A remained relatively stable (PRI + 0.6, VAS + 0.1, PPI + 0.1); those of Group B slightly rebounded (PRI + 1.2, VAS + 0.3, PPI + 0.2); and those of Group C significantly rebounded (PRI + 7.6, VAS + 1.5, PPI + 0.4). Statistical analysis confirmed the significant difference between the groups (*p* < 0.05), indicating that Group A exhibited faster, broader and more permanent pain relief.

### 3.3 Comparison of lumbar spine function improvement among the three groups at different time points

There were no significant differences in anterior flexion, posterior extension, left lateral flexion or right lateral flexion functions among the three groups of patients before treatment (T0) (*d* < 0.2, *p* > 0.05). Lumbar spine function (forwards flexion, backwards extension, and lateral flexion) improved with treatment time in all three groups (all *p* < 0.05), with the greatest and sustained improvement in Group A. In 21 days of treatment (AT21), the effect sizes between Group A and Group C were 1.63 for anterior flexion, 0.99 for posterior extension, 0.79 for left lateral flexion, and 0.74 for right lateral flexion. The effect sizes between Group A and Group B were 0.74, 0.44, 0.35, and 0.40 for the same respective movements. Forwards flexion mobility in Group A had improved to 48.6 ± 4.6° (a 30.3% increase from baseline), and backwards and lateral flexion mobility had increased by 38.9 and >50%, respectively (Table 3). A comparison of the groups revealed that Group A had significantly better functional recovery than Groups B and C did at all follow-up time points (both *p* < 0.05). According to Table 3, the rate of pain improvement was faster in Group A than in the other groups. Group B improved faster than did Group C but was still exhibited slower improvement than that in Group A, and Group C entered the functional plateau after AT14.

**Table 3:** Comparison of lumbar spine function improvement among the three groups at different time points.

Lumbar spine function	Time	Group A (*n* = 40)	Group B (*n* = 40)	Group C (*n* = 40)	Cohen’s *d* A vs. B	Cohen’s *d* A vs. C	Cohen’s *d* B vs. C
Anterior flexion (°)	T0	37.3 ± 5.2	37.1 ± 5.4	37.2 ± 5.3	0.04	0.02	−0.02
	AT7	42.6 ± 4.9^bc^	40.8 ± 5.1^c^	38.5 ± 5.3	0.36	0.80	0.44
	AT14	46.3 ± 4.7^bc^	43.2 ± 5.0^c^	39.8 ± 5.2	0.64	1.31	0.67
	AT21	48.6 ± 4.6^bc^	45.1 ± 4.9^c^	40.7 ± 5.1	0.74	1.63	0.88
Posterior extension (°)	T0	16.2 ± 4.1	16.0 ± 4.3	16.1 ± 4.2	0.05	0.02	−0.02
	AT7	19.5 ± 3.8^bc^	18.2 ± 4.0^c^	17.3 ± 4.1	0.33	0.56	0.22
	AT14	21.8 ± 3.5^bc^	20.1 ± 3.7^c^	18.2 ± 3.9	0.47	0.97	0.50
	AT21	22.5 ± 3.7^bc^	20.9 ± 3.6^c^	18.8 ± 3.8	0.44	0.99	0.57
Left lateral flexion (°)	T0	9.9 ± 5.0	10.1 ± 5.2	10.0 ± 5.1	−0.04	−0.02	0.02
	AT7	12.3 ± 4.4^bc^	11.5 ± 4.6^c^	10.8 ± 4.7	0.18	0.33	0.15
	AT14	14.1 ± 4.0^bc^	13.0 ± 4.3^c^	11.5 ± 4.5	0.27	0.61	0.34
	AT21	15.2 ± 3.6^bc^	13.9 ± 3.9^c^	12.1 ± 4.2	0.35	0.79	0.45
Right lateral flexion (°)	T0	10.2 ± 5.2	10.4 ± 5.4	10.3 ± 5.3	−0.04	−0.02	0.02
	AT7	12.8 ± 4.5^bc^	11.9 ± 4.7^c^	11.2 ± 4.8	0.20	0.34	0.15
	AT14	14.6 ± 4.1^bc^	13.3 ± 4.4^c^	12.0 ± 4.6	0.31	0.60	0.29
	AT21	15.8 ± 3.8^bc^	14.2 ± 4.2^c^	12.7 ± 4.5	0.40	0.74	0.35

vs. Group B, ^b^*p* < 0.05; vs Group C, ^c^*p* < 0.05. All *p*-values for time, between groups, and time × group interactions were <0.05.

### 3.4 Comparison of the improvement in mood disorders and sleep quality at different time points among the three groups

There were no significant differences in anxiety (HADS-A), depression (HADS-D) or sleep quality (PSQI) scores among the three groups before treatment (*d* < 0.2, *p* > 0.01), indicating comparable baseline levels (Table 4). The analysis of differences between groups revealed that the difference between Group A and Groups B and C was statistically significant from the 7th day of treatment. Specifically, at AT7, the effect sizes for comparisons between Group A and Group C were −0.59 for anxiety (HADS-A), −0.29 for depression (HADS-D), and −0.40 for sleep quality (PSQI). In contrast, the effect sizes between Group A and Group B were −0.39, −0.19, and −0.25 for the same indicators, respectively. These findings suggest that Group A exhibited significantly better early improvements in psychological well-being and sleep quality compared to both Groups B and C (*p* < 0.05) and the difference gradually increased over time. During the treatment and follow-up time (AT7–AT90), Group A demonstrated significant and long-lasting improvements: at 90 days (AT90), anxiety scores decreased by 41.2% from baseline (6.0 ± 1.9 vs. 10.2 ± 3.1), depression scores decreased by 25.3% (6.8 ± 2.0 vs. 9.1 ± 3.0), and sleep quality scores improved by 44.9% (8.6 ± 2.1 vs. 15.6 ± 3.4). All of these indicators were significantly better in Group A than in Groups B and C (both *p* < 0.05). Notably, the improvement in Group A was characterized by a continuous gradual progression, which remained stable after the end of the 21-day course of treatment and up to the 90th day follow-up date (AT21–AT90); Group B had a slight rebound of symptoms after discontinuation of the treatment; and Group C had a symptom rebound phenomenon after the 21-day discontinuation of the drug.

**Table 4:** Comparison of the improvement in mood disorders and sleep quality at different time points among the three groups.

Evaluation indicators	Time	Group A (*n* = 40)	Group B (*n* = 40)	Group C (*n* = 40)	Cohen’s *d* A vs. B	Cohen’s *d* A vs. C	Cohen’s *d* B vs. C
HADS-A (anxiety)	T0	10.2 ± 3.1	10.3 ± 3.2	10.3 ± 3.2	−0.03	−0.03	0.00
	AT7	8.1 ± 2.7^bc^	9.2 ± 2.9^c^	9.8 ± 3.0	−0.39	−0.59	−0.20
	AT14	7.0 ± 2.3^bc^	8.5 ± 2.6^c^	9.5 ± 2.8	−0.61	−0.98	−0.37
	AT21	6.3 ± 2.0^bc^	7.1 ± 2.1^c^	8.3 ± 2.2	−0.39	−0.95	−0.56
	AT90	6.0 ± 1.9^bc^	7.0 ± 2.0^c^	9.4 ± 2.1	−0.51	−1.70	−1.17
HADS-D (depression)	T0	9.1 ± 3.0	9.0 ± 3.1	9.0 ± 3.1	0.03	0.03	0.00
	AT7	8.0 ± 2.6^bc^	8.5 ± 2.8^c^	8.8 ± 2.9	−0.19	−0.29	−0.11
	AT14	7.5 ± 2.4^bc^	8.0 ± 2.5^c^	8.6 ± 2.7	−0.20	−0.43	−0.23
	AT21	7.2 ± 2.1^bc^	7.4 ± 2.2^c^	8.1 ± 2.3	−0.09	−0.41	−0.31
	AT90	6.8 ± 2.0^bc^	7.4 ± 2.1^c^	8.5 ± 2.2	−0.29	−0.80	−0.51
PSQI (sleep quality)	T0	15.6 ± 3.4	15.5 ± 3.5	15.5 ± 3.5	0.03	0.03	0.00
	AT7	12.8 ± 3.1^bc^	13.6 ± 3.3^c^	14.1 ± 3.4	−0.25	−0.40	−0.15
	AT14	10.9 ± 2.8^bc^	12.3 ± 3.0^c^	13.8 ± 3.3	−0.48	−0.95	−0.48
	AT21	9.7 ± 2.3^bc^	10.6 ± 2.8^c^	12.5 ± 3.0	−0.35	−1.05	−0.66
	AT90	8.6 ± 2.1^bc^	10.1 ± 2.7^c^	13.5 ± 2.9	−0.62	−1.94	−1.21

vs. Group B, ^b^*p* < 0.05; vs Group C, ^c^*p* < 0.05. All *p*-values for time, between groups, and time × group interactions were <0.05.

### 3.5 Comparison of the Five Fact Mindfulness Questionnaire scores of patients in Group A at different time points

Patients in Group A showed a stepwise increase in FFMQ score with increasing treatment time (*p* < 0.05) (Table 5). These findings indicated that patients in Group A received effective mindfulness meditation treatment.

**Table 5:** Comparison of the Five Fact Mindfulness Questionnaire (FFMQ) scores of patients in Group A at different time points (*n* = 40).

Time	FFMQ score (Group A)	*p*
T0	92 ± 6.5	<0.05
AT7	98 ± 7.1	
AT14	106 ± 4.6	
AT21	112 ± 5.2	

There is a statistically significant difference at different time points (*p* < 0.05).

### 3.6 Changes in TNF-α and IL-1β before and after treatment in the three groups

At the end of the treatment (AT21), Groups A, B and C were able to reduce TNF-α and IL-1β in the serum of patients with lumbar MPS (all *p* < 0.05) (Table 6). Notably, the decrease in TNF-α and IL-1β levels was significantly greater in Group C than in Groups A and B (*p* < 0.05).

**Table 6:** Changes in TNF-α and IL-1β before and after treatment in the three groups.

Outcome	Group (*n* = 40)	Baseline	Day 21	*p*
TNF- α (pg/mL)	A	41.6 ± 4.2	24.5 ± 3.1	<0.05
	B	39.1 ± 4.5	25.5 ± 6.5	<0.05
	C	40.3 ± 4.8	19.8 ± 6.3	<0.05
IL-1 β (pg/mL)	A	69.5 ± 9.1	60.1 ± 6.9	<0.05
	B	68.8 ± 9.2	60.6 ± 9.5	<0.05
	C	70.6 ± 9.5	55.3 ± 8.4	<0.05

There is a statistically significant difference between baseline and Day 21 comparisons in the same group (*p* < 0.05).

## 4 Discussion

This study is the first to validate the synergistic therapeutic effect of ultrasound-guided acupotomy combined with mindfulness meditation therapy on lumbar MPS. This innovative combined treatment program demonstrated significant advantages in pain relief, functional recovery, and improvement of psychological and sleep status, providing new ideas for the clinical treatment of lumbar MPS.

**Figure 3:**
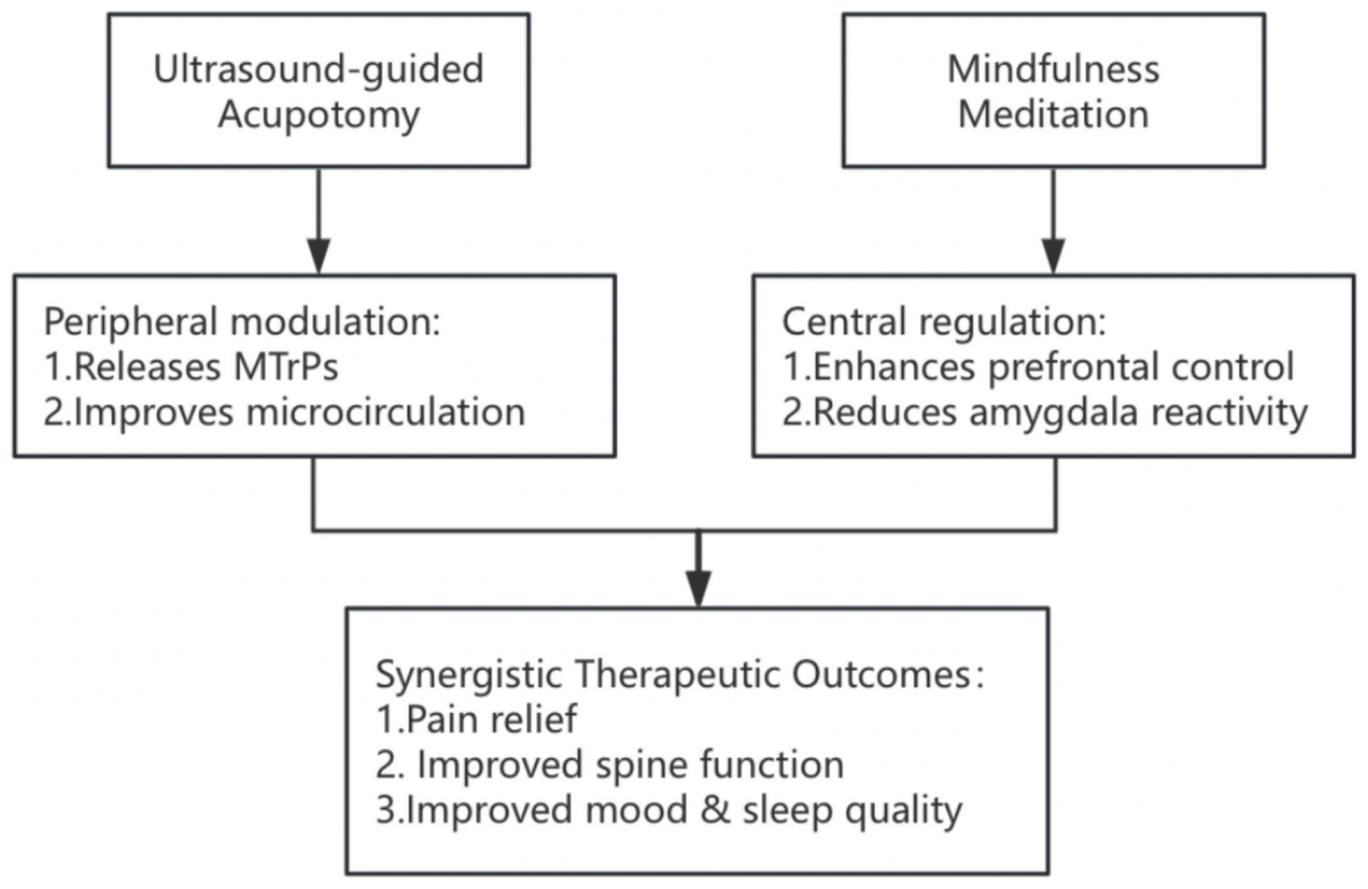
Framework of peripheral–central regulatory mechanisms in combined treatment of lumbar MPS.

In terms of pain relief, ultrasound-guided acupotomy combined with mindfulness meditation showed rapid onset of action, significant improvement, and long-lasting efficacy. This efficacy may result from the synergistic effect of the two treatment modalities: ultrasound-guided acupotomy directly reduces local mechanical tension and improves muscle-fascial function by precisely loosening the myofascial trigger points, whereas mindfulness meditation reduces central sensitization by modulating the pain modulation networks in the anterior cingulate gyrus cortex and insula (Figure 3). This finding echoes the results of previous studies: a systematic evaluation by Zhao et al. (21) confirmed that acupotomy effectively relieves back pain by loosening myofascial trigger points, whereas a randomized controlled trial by Chen et al. (22) revealed that ultrasound-guided acupotomy significantly improved pain symptoms in patients with osteoarthritis of the knee. Moreover, Fedeli et al. (23) reported that mindfulness meditation significantly reduced pain perception in chronic pain patients by modulating the activity of the anterior cingulate cortex. The novel contribution of the present study is the combination of the advantages of these two treatment modalities to achieve better pain relief. It should be noted that the hypothesized mechanisms of action, namely peripheral modulation through acupotomy and central regulation via mindfulness meditation, are inferred from previous literature. Future studies could incorporate neurophysiological techniques, such as functional magnetic resonance imaging (fMRI), electroencephalography (EEG), functional near-infrared spectroscopy (fNIRS), or heart rate variability analysis (HRV), to objectively assess brain activity, autonomic function, and pain-related neural circuits. These tools will help to provide stronger evidence for validating the hypothesized mechanisms for the “peripheral relaxation-central regulation” of the combined interventions.

In terms of lumbar spine function improvement, patients in Group A showed greater improvement in forwards flexion, backwards extension and lateral flexion mobility than did those in Group B and were significantly improvements compared with Group C. This result is similar to the findings of Yanling et al. (24) study on the treatment of thoracodorsal myofascial pain with acupotomy, which confirmed that, compared with drug use, acupotomy significantly improved cervical spine mobility. Moreover, Spani et al. (25) fMRI study confirmed that meditation training could enhance the functional connectivity between the primary motor cortex (M1 area) and the supplementary motor area, and fMRI revealed a significant increase in neurological efficiency during the preparation phase of movement. Perez-Diaz et al. (26) used the Sahaja Yoga meditation training method and reported that training could improve white matter fiber connectivity and enhance motor coordination, suggesting that mindfulness meditation can substantially improve brain function and thus motor status. Therefore, the improvement effect of the combined treatment not only comes from the mechanical loosening effect of acupotomy but also includes the regulatory effect of mindfulness meditation on patients’ movement patterns, and mindfulness meditation effectively promotes the relearning of correct movement patterns by enhancing awareness of abnormal postures and improving brain function, thus effectively improving lumbar MPS in the long term.

In terms of psychological state and sleep quality improvement, Group A showed significant advantages. Although acupotomy alone can directly alleviate pain-related sleep disorders through pain relief, it has some limitations in terms of effectively intervening in patients’ pain perception and emotional state at the psychological level. Therefore, the combination of mindfulness meditation therapy can compensate for the shortcomings of acupotomy treatment in psychological intervention and provide a more comprehensive treatment program for patients. Mindfulness meditation training can weaken the functional connection between the posterior cingulate cortex (PCC) and the medial prefrontal lobe (mPFC), and this decoupling is directly related to a reduction in negative emotions, which can improve symptoms of anxiety and depression (27–30). Yang et al. (31) reported that after 8 weeks of stress reduction training with mindfulness meditation, the strength of internal connections in the DMN of anxiety patients was reduced by 32%; additionally, mindfulness meditation can regulate the function of the autonomic nervous system, increase parasympathetic tone, and further improve the quality of sleep (32, 33). Combined with the trend of the PSQI scores in this study, the improvement in sleep quality and mood disorders in the combined treatment group was characterized by gradual progression, which continued to progress even after the end of treatment, which coincided with the time course of the neuroplasticity changes induced by mindfulness meditation training, suggesting that the mood disorders and sleep quality of patients could be improved by effective mindfulness meditation training. The unique advantage of the combined treatment group is that acupotomy can improve the pain of patients with localized symptoms in a short period of time, mindfulness meditation therapy can improve the psychological state of patients in a long period of time, and the combination of the two breaks the vicious cycle of “pain–emotion–sleep,” which is better and lasts for a longer period of time than does pure acupotomy therapy and drug therapy.

Regarding the serum, the local microenvironment of MTrPs is characterized by a significant increase in the levels of proinflammatory mediators, including inflammatory cytokines (e.g., TNF-α and IL-1β), neuropeptides (e.g., substance P), and catecholamines (34–36). In this study, the celecoxib group exhibited the most significant decrease in serum TNF-α and IL-1β levels among the three groups. This result may be attributed to its pharmacological mechanism as a selective cyclooxygenase-2 (COX-2) inhibitor. By selectively inhibiting COX-2, celecoxib blocks the conversion of arachidonic acid into prostaglandin E2 (PGE2), a key mediator in the inflammatory cascade. This inhibition not only alleviates peripheral inflammatory responses but also downregulates the systemic expression of proinflammatory cytokines, including TNF-α and IL-1β (37). In contrast to the direct anti-inflammatory action of celecoxib, acupotomy and mindfulness meditation may reduce inflammation through indirect mechanisms. These include improving microcirculation and regulating neuroendocrine pathways, which in turn help lower the release of proinflammatory mediators. Although both interventions demonstrated anti-inflammatory effects, celecoxib showed a more pronounced inhibitory effect on TNF-α and IL-1β levels. Beyond the direct effects of celecoxib, this study also found that both Group A and Group B significantly reduced serum TNF-α and IL-1β levels. This may be due to acupotomy’s ability to release fascial adhesions and enhance local microcirculation, thereby reducing the accumulation of inflammatory substances. Additionally, mindfulness meditation in Group A may have further enhanced the anti-inflammatory response by modulating central proinflammatory signaling and suppressing neurogenic inflammation. This non-pharmacological approach offers a safer and more versatile treatment option for patients who are not candidates for non-steroidal drugs (NSAIDs).

Clinically, this combination therapy also has synergistic advantages, as the immediate symptomatic improvement produced by acupotomy treatment significantly enhances patients’ adherence to mindfulness meditation, and the self-management skills fostered by mindfulness meditation prolong the maintenance of acupotomy treatment, creating a virtuous circle. A review by Ploesser and Martin (38) emphasized that this integrated biopsychosocial intervention model is particularly suited to the long-term management of chronic pain.

This study also has several limitations. First, the follow-up duration of 90 days is still insufficient for evaluating long-term efficacy; in the future, the follow-up time can be extended, and the follow-up density can be increased to track the trajectory of efficacy changes more accurately. Additionally, the detection of inflammatory indicators is relatively limited, and only two inflammatory factors, TNF-α and IL-1β, were detected in the present study. Such a limited scope of detection may not comprehensively reflect the inflammatory network of lumbar myofascial pain syndromes. A comprehensive analysis of inflammation- and metabolism-related biomarkers via a multiomics approach could be considered in the future. Due to the specific nature of the intervention, it was not feasible to blind participants and treatment providers, which may have introduced detection bias. Although outcome assessors and data managers were blinded, bias in patient-reported outcomes could not be completely excluded. Future studies should consider using objective assessment metrics or sham intervention control designs to minimize bias.

In conclusion, this study is the first to confirm that ultrasound-guided acupotomy combined with mindfulness meditation therapy has significant advantages in the treatment of lumbar MPS. Through the dual mechanism of “peripheral relaxation–central regulation,” this integrated treatment mode not only rapidly relieves pain and improves lumbar spine function but also improves the mood disorders and sleep quality of patients and improves lumbar MPS from the root. These findings underscore the clinical value of combining ultrasound-guided acupotomy with mindfulness meditation, offering a comprehensive and sustainable approach to treating chronic pain.

## Data Availability

The original contributions presented in the study are included in the article/supplementary material, further inquiries can be directed to the corresponding author.

## References

[1] Dach F Ferreira KS. Treating myofascial pain with dry needling: a systematic review for the best evidence-based practices in low back pain. Arq Neuropsiquiatr. (2023) 81:1169–78. doi: 10.1055/s-0043-1777731, PMID: 38157883 PMC10756779

[2] Lara-Palomo IC Gil-Martínez E Antequera-Soler E Castro-Sánchez AM Fernández-Sánchez M García-López H. Electrical dry needling versus conventional physiotherapy in the treatment of active and latent myofascial trigger points in patients with nonspecific chronic low back pain. Trials. (2022) 23:238. doi: 10.1186/s13063-022-06179-y, PMID: 35346331 PMC8961901

[3] Booth J Moseley GL Schiltenwolf M Cashin A Davies M Hübscher M. Exercise for chronic musculoskeletal pain: a biopsychosocial approach. Musculoskeletal Care. (2017) 15:413–21. doi: 10.1002/msc.1191, PMID: 28371175

[4] Mao JJ Liou KT Baser RE Bao T Panageas KS Romero SAD. Effectiveness of electroacupuncture or auricular acupuncture vs usual care for chronic musculoskeletal pain among cancer survivors: the PEACE randomized clinical trial. JAMA Oncol. (2021) 7:720–7. doi: 10.1001/jamaoncol.2021.0310, PMID: 33734288 PMC7974834

[5] Giamberardino MA Affaitati G Fabrizio A Costantini R. Myofascial pain syndromes and their evaluation. Best Pract Res Clin Rheumatol. (2011) 25:185–98. doi: 10.1016/j.berh.2011.01.002, PMID: 22094195

[6] Rahangdale A Ferraro J. Assessing comorbid PTSD, depression, and anxiety in fibromyalgia patients: a retrospective observational study. BMC Psychiatry. (2025) 25:444. doi: 10.1186/s12888-025-06708-4, PMID: 40312303 PMC12044831

[7] Thottungal A Kumar P Bhaskar A. Interventions for myofascial pain syndrome in cancer pain: recent advances: why, when, where and how. Curr Opin Support Palliat Care. (2019) 13:262–9. doi: 10.1097/SPC.0000000000000446, PMID: 31348012

[8] Xiao J Cao BY Xie Z Ji YX Zhao XL Yang HJ. Clinical efficacy of electromagnetic field therapy combined with traditional Chinese pain-reducing paste in myofascial pain syndrome. World J Clin Cases. (2022) 10:11753–65. doi: 10.12998/wjcc.v10.i32.11753, PMID: 36405282 PMC9669869

[9] Dommerholt J Hooks T Finnegan M Grieve R. A critical overview of the current myofascial pain literature—March 2016. J Bodyw Mov Ther. (2016) 20:397–408. doi: 10.1016/j.jbmt.2016.02.015, PMID: 27210859

[10] Kwon CY Yoon SH Lee B. Clinical effectiveness and safety of acupotomy: an overview of systematic reviews. Complement Ther Clin Pract. (2019) 36:142–52. doi: 10.1016/j.ctcp.2019.07.002, PMID: 31383431

[11] Sierpina VS Frenkel MA. Acupuncture: a clinical review. South Med J. (2005) 98:330–7. doi: 10.1097/01.SMJ.0000140834.30654.0F, PMID: 15813160

[12] Qiu Z Jia Y Shen Y Zhou Q Sun X Zhu X. Acupotomy by an ultrasound-guided technique: a protocol for a systematic review. Medicine. (2019) 98:e17398. doi: 10.1097/MD.0000000000017398, PMID: 31626093 PMC6824636

[13] Liang YS Chen LY Cui YY Du CX Xu YX Yin LH. Ultrasound-guided acupotomy for trigger finger: a systematic review and meta-analysis. J Orthop Surg Res. (2023) 18:678. doi: 10.1186/s13018-023-04127-3, PMID: 37705066 PMC10498646

[14] Fox KCR Nijeboer S Dixon ML Floman JL Ellamil M Rumak SP. Is meditation associated with altered brain structure? A systematic review and meta-analysis of morphometric neuroimaging in meditation practitioners. Neurosci Biobehav Rev. (2014) 43:48–73. doi: 10.1016/j.neubiorev.2014.03.016, PMID: 24705269

[15] Khalsa DS. Stress, meditation, and Alzheimer’s disease prevention: where the evidence stands. J Alzheimer’s Dis. (2015) 48:1–12. doi: 10.3233/JAD-142766, PMID: 26445019 PMC4923750

[16] Zollars I Poirier TI Pailden J. Effects of mindfulness meditation on mindfulness, mental well-being, and perceived stress. Curr Pharm Teach Learn. (2019) 11:1022–8. doi: 10.1016/j.cptl.2019.06.005, PMID: 31685171

[17] Brandmeyer T Delorme A Wahbeh H. The neuroscience of meditation: classification, phenomenology, correlates, and mechanisms. Prog Brain Res. (2019) 244:1–29. doi: 10.1016/bs.pbr.2018.10.020244 30732832

[18] Tang YY Hölzel BK Posner MI. The neuroscience of mindfulness meditation. Nat Rev Neurosci. (2015) 16:213–25. doi: 10.1038/nrn3916, PMID: 25783612

[19] Kabat-Zinn J. An outpatient program in behavioral medicine for chronic pain patients based on the practice of mindfulness meditation: theoretical considerations and preliminary results. Gen Hosp Psychiatry. (1982) 4:33–47. doi: 10.1016/0163-8343(82)90026-3, PMID: 7042457

[20] Hilton L Hempel S Ewing BA Apaydin E Xenakis L Newberry S. Mindfulness meditation for chronic pain: systematic review and meta-analysis. Ann Behav Med. (2017) 51:199–213. doi: 10.1007/s12160-016-9844-2, PMID: 27658913 PMC5368208

[21] Zhao Y Yang Y Kong X Liu J Hong J Huang X. Needling trigger points for treating myofascial pain syndrome: a systematic review and meta-analysis. Complement Ther Clin Pract. (2025) 59:101978. doi: 10.1016/j.ctcp.2025.101978, PMID: 40199184

[22] Chen C Liu D Guo SX Chen B Wang SY Chen PH. Clinical effects of ultrasound-guided acupotomy in knee osteoarthritis treatment. J Vis Exp. (2024) 26:e66587. doi: 10.3791/66587, PMID: 38738888

[23] Fedeli D Ciullo G Demichelis G Medina Carrion JP Bruzzone MG Ciusani E. Longitudinal neurofunctional changes in medication overuse headache patients after mindfulness practice in a randomized controlled trial (the MIND-CM study). J Headache Pain. (2024) 25:97. doi: 10.1186/s10194-024-01803-5, PMID: 38858629 PMC11165872

[24] Yanling Z Hong L Wang C Nie Y Xiong Y Zheng Z. Efficacy and safety of ultrasound-guided acupotomy versus celecoxib in patients with thoracodorsal myofascial pain syndrome: a randomized controlled trial. J Integr Complement Med. (2024) 30:986–94. doi: 10.1089/jicm.2023.0490, PMID: 38770602

[25] Spani F Carducci F Piervincenzi C Ben-Soussan TD Mallio CA Quattrocchi CC. Assessing brain neuroplasticity: surface morphometric analysis of cortical changes induced by Quadrato motor training. J Anat. (2025) 246:757–69. doi: 10.1111/joa.14104, PMID: 38924527 PMC11996713

[26] Perez-Diaz O Góngora D González-Mora JL Rubia K Barrós-Loscertales A Hernández SE. Enhanced amygdala–anterior cingulate white matter structural connectivity in Sahaja yoga meditators. PLoS One. (2024) 19:e0301283. doi: 10.1371/journal.pone.0301283, PMID: 38547155 PMC10977753

[27] Joss D Teicher MH Lazar SW. Temporal dynamics and long-term effects of a mindfulness-based intervention for young adults with adverse childhood experiences. Mindfulness. (2024) 15:2245–61. doi: 10.1007/s12671-024-02439-x, PMID: 40160902 PMC11951444

[28] García-Pérez L Atencia-Rodriguez ME Cepero-González M Padial-Ruz R. Effectiveness of physical activity, mindfulness and mind-body therapies in improving mental health of university students: a systematic review of RCTS. J Am Coll Heal. (2025):1–16. doi: 10.1080/07448481.2025.2492174 40262195

[29] Kral TRA Lapate RC Imhoff-Smith T Patsenko E Grupe DW Goldman R. Long-term meditation training is associated with enhanced subjective attention and stronger posterior cingulate–rostrolateral prefrontal cortex resting connectivity. J Cogn Neurosci. (2022) 34:1576–89. doi: 10.1162/jocn_a_01881, PMID: 35704552 PMC9357181

[30] Brewer JA Garrison KA. The posterior cingulate cortex as a plausible mechanistic target of meditation: findings from neuroimaging. Ann N Y Acad Sci. (2014) 1307:19–27. doi: 10.1111/nyas.12246, PMID: 24033438

[31] Yang CC Barrós-Loscertales A Li M Pinazo D Borchardt V Ávila C. Alterations in brain structure and amplitude of low-frequency after 8 weeks of mindfulness meditation training in meditation-naïve subjects. Sci Rep. (2019) 9:10977. doi: 10.1038/s41598-019-47470-4, PMID: 31358842 PMC6662752

[32] Li Y Tang J Chen G. The effect of meditation-based mind-body interventions on older adults with poor sleep quality: a meta-analysis of randomized controlled trials. Behav Sleep Med. (2025) 23:341–59. doi: 10.1080/15402002.2025.2475911, PMID: 40100065

[33] Kim S Kang J. Effects of virtual reality meditation on sleep and delirium in ICU patients: a randomized controlled trial. Comput Inform Nurs. (2025) 43:e01307. doi: 10.1097/CIN.0000000000001307, PMID: 40194914 PMC12321352

[34] Hsieh YL Hong CZ Liu SY Chou LW Yang CC. Acupuncture at distant myofascial trigger spots enhances endogenous opioids in rabbits: a possible mechanism for managing myofascial pain. Acupunct Med. (2016) 34:302–9. doi: 10.1136/acupmed-2015-011026, PMID: 27143259

[35] Shah JP Phillips TM Danoff JV Gerber LH. An *in vivo* microanalytical technique for measuring the local biochemical milieu of human skeletal muscle. J Appl Physiol. (2005) 99:1977–84. doi: 10.1152/japplphysiol.00419.2005, PMID: 16037403

[36] Yu S Su H Lu J Zhao F Jiang F. Combined T2 mapping and diffusion tensor imaging: a sensitive tool to assess myofascial trigger points in a rat model. J Pain Res. (2021) 14:1721–31. doi: 10.2147/JPR.S313966, PMID: 34163230 PMC8214538

[37] Lv H Li Z Hu T Wang Y Wu J Li Y. The shear wave elastic modulus and the increased nuclear factor kappa B (NF-kB/p65) and cyclooxygenase-2 (COX-2) expression in the area of myofascial trigger points activated in a rat model by blunt trauma to the vastus medialis. J Biomech. (2018) 66:44–50. doi: 10.1016/j.jbiomech.2017.10.028, PMID: 29137729

[38] Ploesser M Martin D. Mechanism of action of mindfulness-based interventions for pain relief—a systematic review. J Integr Complement Med. (2024) 30:1162–78. doi: 10.1089/jicm.2023.0328, PMID: 39042592 PMC11659456

